# Understanding inherited cardiomyopathies: clinical aspects and genetic determinants

**DOI:** 10.1515/medgen-2025-2007

**Published:** 2025-04-08

**Authors:** Gökhan Yigit, Silke Kaulfuß, Bernd Wollnik

**Affiliations:** Institute of Human Genetics University Medical Center Göttingen Heinrich-Düker-Weg 12 37073 Göttingen Germany; Institute of Human Genetics University Medical Center Göttingen Heinrich-Düker-Weg 12 37073 Göttingen Germany; Georg-August University Göttingen Institute of Human Genetics Heinrich-Düker-Weg 12 37073 Göttingen Germany

**Keywords:** Heritable cardiomyopathies, hypertrophic cardiomyopathy, dilated cardiomyopathy, cardiogenetics, genetic testing

## Abstract

Cardiomyopathies (CMs) are a clinically heterogeneous group of cardiovascular diseases characterized by structural and functional abnormalities of the heart muscle in the absence of coronary artery disease, hypertension, valve disease, or congenital heart disease as a leading cause. The phenotypic spectrum of CMs ranges from silent heart failure to symptomatic heart failure and sudden cardiac death, and CMs are one of the leading causes of cardiovascular morbidity worldwide. CMs are highly heritable, although a clear distinction between inherited and acquired forms remains challenging, particularly due to observed incomplete penetrance and variable expressivity of inherited CMs. Based on their specific morphological phenotypes and functional characteristics, CMs can be divided into at least 5 different subgroups: hypertrophic cardiomyopathy (HCM), dilated cardiomyopathy (DCM), arrhythmogenic cardiomyopathy (ACM), restrictive cardiomyopathy (RCM), and (left ventricular) non-compaction cardiomyopathy (LVNC), which show both clinical as well as genetic overlap. Since the identification of pathogenic variants in *MYH7* as a genetic cause of HCM in 1990, enormous progress has been made in understanding genetic factors contributing to cardiomyopathies. Currently, over 100 genes have been associated with at least one of the CM subtypes, providing a deeper understanding of the cellular basis of genetic heart failure syndromes, unveiling new insights into the molecular biology of heart function in both health and disease, and, thereby, facilitating the development of novel therapeutic strategies and personalized treatment approaches.

## Introduction

Cardiomyopathies (CMs) refer to a diverse spectrum of isolated or syndromic diseases that affect the myocardium, impairing the structure and functional integrity of the heart muscle. In addition to exogenous factors such as viral infections and toxins, CMs can arise from germline mutations in a large array of distinct genes implicated in essential functional and structural processes in cardiomyocytes. Inherited CMs occur with an incidence of 1 in 200 to 500 individuals and they comprise common presentations like hypertrophic (HCM) and dilated cardiomyopathy (DCM) as well as rare and infrequent forms including arrhythmogenic cardiomyopathy (ACM), restrictive cardiomyopathy (RCM), and (left ventricular) non-compaction cardiomyopathy (LVNC) [1]. Notably, causative variants within the same gene have been observed to be associated with distinct subtypes of CMs, suggesting an overlapping molecular pathogenesis of these conditions. The clinical characteristics and molecular determinants of the two main forms of CMs, HCM and DCM, are comprehensively described in this review.

## Hypertrophic cardiomyopathy

**Figure 1:**
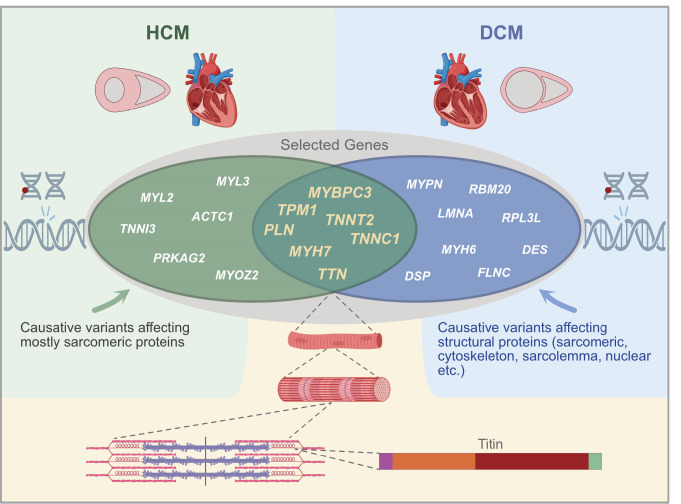
**HCM and DCM, the most prevalent genetic cardiomyopathies.** HCM is morphologically characterized by left ventricular hypertrophy, DCM by ventricular enlargement. The inheritance pattern for monogenic forms of HCM and DCM is mainly autosomal dominant, but all other patterns of inheritance including mitochondrial inheritance have also been described. Selected, most frequently implicated genes. Sarcomeric mutations may underlie both HCM and DCM, including variants in *TTN*, encoding the largest sarcomeric protein, titin. Schematic domain structure of titin protein, adapted from [68]; purple = Z disc, orange = I-band, red = A-band, green = M-band. Created with BioRender.com.

With an estimated worldwide prevalence of 1:500 to 1:1,000 in the general population, HCM (including hypertrophic obstructive cardiomyopathy (HOCM)) is one of the most prevalent inherited cardiomyopathy globally [2, 3]. This makes it a significant contributor to atrial fibrillation and/or heart failure, particularly in young and previously asymptomatic patients. Clinically, HCM can be diagnosed based on morphological changes in the left ventricular (LV) wall, specifically hypertrophy without dilatation of the chamber and/or other cardiac or systemic diseases [1]. Cardiac tissue of patients with HCM exhibit histological changes, such as myofiber disarray and often interstitial fibrosis. Additionally, patients may present with mild right ventricular hypertrophy, morphologic anomalies of the mitral valve, myocardial fibrosis, and electrocardiographic abnormalities [4, 5]. The clinical manifestations of HCM vary widely depending on the severity of left ventricular ejection obstruction caused by morphological changes. These manifestations can range from asymptomatic heart failure, mild or severe arrhythmias, and symptomatic heart failure to sudden cardiac death [6]. Genetically, HCM is a heterogeneous disorder as indicated by the identification of numerous pathogenic variants in nearly 30 genes to date [7–9]. The majority of known genetic causes of HCM follow an autosomal dominant pattern of inheritance, but cases and types with autosomal recessive, X-linked and mitochondrial inheritance have also been described and clinically associated with an earlier disease onset and often more severe clinical presentation and progression [10–13]. Since the first pathogenic variants in *MYH7* encoding the beta-myosin heavy chain were identified in 1990, several additional genes coding for sarcomeric components have been found to be mutated, leading to the classification of HCM as a disease of the sarcomere. These genes encompass the sarcomeric components *myosin-binding protein 3* (*MYBP3*), *troponin T* (*TNNT*), *troponin I* (*TNNI*), and *tropomyosin* (*TPM1*), collectively accounting for approximately 90 % of HCM cases, for which a molecular genetic diagnosis could be established [14, 15] (Figure 1). Pathogenic variants in genes encoding sarcomeric proteins can disrupt contractile mechanisms and calcium homeostasis within the sarcomere, reducing overall sarcomeric power and inducing a remodeling process that results in cardiomyocyte hypertrophy and the characteristic clinical features of HCM. Over the past few years, particularly due to the advent of NGS-based technologies and applications, pathogenic variants in an increasing number of non-sarcomeric genes have been identified and associated with HCM. Examples include *ALPK3* (*alpha kinase 3*), *FHOD3* (*formin homology 2 domain containing 3*), and* JPH2* (*junctophilin 2*), which have expanded the definition of HCM beyond a sarcomeric disease to encompass other cellular processes [9, 16, 17]. HCM can also occur as part of a congenital disorder or metabolic disease including lysosomal and glycogen storage diseases (e.g. Danon disease, Fabry disease, and Pompe disease), RASopathies (e.g. Noonan syndrome), and amyloidosis that manifest with cardiac changes [18, 19]. These syndromic types of HCM, which can exhibit morphological cardiac alterations that closely resemble classical forms of sarcomeric HCM, may evade specific syndrome diagnosis in early childhood due to the mild presentation of extracardiac symptoms at the time of HCM diagnosis.

## Dilated cardiomyopathy

Dilated cardiomyopathy (DCM) is a cardiac condition characterized by the dilatation of the left ventricle in the absence of significant coronary artery, hypertensive, or valvular heart disease, which leads to reduced systolic and contractile function of the heart. With an estimated prevalence of 1 in 250, it is one of the most prevalent forms of cardiomyopathies and the leading cause of cardiac transplantation in both children and adults [18, 19]. The majority of DCM cases are classified as sporadic; however, approximately 20 to 30 % of cases exhibit a positive family history and likely heritable pattern. Similar to HCM, familial forms of DCM display pronounced genetic heterogeneity, and are inherited mainly autosomal dominantly, though autosomal recessive, X-linked, and mitochondrial inheritance patterns are also observed. Notably, mitochondrial inheritance is frequently associated with extracardiac manifestation of disease, including skeletal muscles, brain, eyes and ears. Pathogenic variants in a large set of genes have been associated with DCM, primarily in genes encoding components of the sarcomere, the Z-disc, the desmosome, or the nuclear envelope [20–25]. Despite advance in genetic testing, diagnostic yields remain low, particularly for non-syndromic cases [26]. Genetic counselling and risk assessment is further complicated by the highly variable penetrance of identified variants and the fact that variants within the same genes can cause distinct cardiomyopathy-related phenotypes. Furthermore, DCM can also manifest as part of a congenital myopathy, as evidenced in patients with X-linked dystrophinopathies, such as Duchenne/Becker muscular dystrophy. In such instances, pathogenic variants in the underlying genes can affect both skeletal and cardiac muscle tissues, and, consequently, patients, including female carriers of pathogenic variants, may exhibit muscle weakness along with myocardial dysfunction and heart failure [27–29]. Currently, over 100 distinct genes have been associated with DCM [30; OMIM – Online Mendelian Inheritance in Man 2024]. Among these, disease-causative truncating variants in the *titin* (*TTN*) gene are the most prevalent causative variations in patients with non-syndromic DCM, accounting for 15–25 % of all DCM cases (see below) [31–33]. Additional significant genetic causes of DCM include variants in *lamin A/C* (*LMNA*), which are identified in approximately 5 % of DCM cases, *RNA-binding motif-20* (*RBM20*), *filamin C* (*FLCN*), *desmin* (*DES*), and *desmoplakin* (*DSP*) [21, 34, 35]. Given the clinical pathomechanistical overlap with other cardiomyopathies, such as HCM, additional core genes that are associated with DCM include *MYH7*, *MYBPC3*, *TNNC1*, *TNNT2*, and *TPM1* (Figure 1).

## Challenges in classifying *TTN* variants: a diagnostic perspective

*TTN* contains 364 exons and encodes the largest protein of the human body. After myosin and actin, TTN is the most abundant protein in striated muscle cells of vertebrates, spanning half the sarcomere and serving as a biological spring [36]. It is composed of four main structural regions: (i) the N-terminal Z-disc, which anchors TTN to the sarcomeric disc and is critical for myofibril assembly and stability, (ii) the central I-band, which contains a variable number of immunoglobulin-repeats and confers elasticity to TTN, (iii) the A-band, which serves as a stable anchor for myosin binding and is crucial for maintaining the structural integrity of thick filaments, and (iiii) the C-terminal M-band, which encompasses a putative serine/threonine kinase domain and is involved in signaling and scaffolding processes. *TTN* pre-mRNA undergoes extensive alternative splicing, primarily affecting exons coding for the I-band region [37–39]. Consequently, this process generates a substantial variety of different isoforms that confer tissue-specific mechanical properties to both skeletal and cardiac muscle. In cardiac muscle, two major classes of isoforms, N2B and N2AB, are expressed, which are predominantly generated by alternative splicing in the exons encoding the I-band region [37, 40, 41]. Interestingly, alterations in alternative splicing, which impair the generation of cardiac-specific *TTN* isoforms, such as those caused by pathogenic variants in the splicing factor RBM20, have been associated with the development of DCM in humans [42]. Truncating variants in *TTN* are identified in 15 to 25 % of familial and sporadic DCM patients, making *TTN* the most frequently mutated gene in DCM. Still, variant classification for *TTN* remains challenging. Large-scale population studies have indicated that approximately 1–3 % of the general population, who do not exhibit symptoms of DCM or HCM, carry nonsense, frameshift or splice-site variants in *TTN* [31]. This suggests that in addition to a direct mutational effect, other mutational features may also contribute significantly to assessing the pathogenicity of novel *TTN* variants. Indeed, it has been shown that the localization of truncating *TTN* variants within the gene has a severe impact on their pathogenicity. Truncating variants affecting exons coding for the A- and, to a lesser extent, the I-band region of cardiac *TTN* isoforms are more likely to exert a pathogenic effect compared to variants encoding the N-terminal Z-band [43]. Therefore, classifying and interpreting identified variants during routine diagnostic procedures remains a significant challenge. As previously discussed, truncating variants in specific protein regions can be safely classified as pathogenic or likely pathogenic based on ACMG criteria. However, determining the functional consequences of the majority of *TTN* variants detected by NGS-based sequencing approaches presents a more complex challenge. We do find numerous missense variants, intronic variants, small in-frame deletions, and duplications, and truncating variants in variable regions of the protein, which cannot yet be adequately interpreted and are often reported as variants of unknown significance (VUS). To enhance variant interpretation and diagnostic yield, DCM-specific modification of ACMG guidelines were suggested incorporating DCM-specific disease and genetic features for *TTN* and additional DCM-associated genes [44]. Additionally, we strongly recommend performing long-read RNAseq in parallel to DNA testing, which enables the estimation of the effect of *TTN* variants on its RNA composition, thereby increasing the effectiveness of variant classification.

In addition to their role in DCM, causative variants in the *TTN* gene have also been associated with HCM, RCM, ARVC, and, most importantly, atrial fibrillation. Recent studies utilizing data from the UK Biobank have demonstrated that truncating variants in the cardiac *TTN* isoforms substantially elevates the overall risk of atrial fibrillation (AF) and the likelihood of heart failure progression [45, 46].

## Novel genes and mechanisms in inherited CMs – the *RPL3L* example

Within the past few years, advances in NGS-based approaches, such as whole-exome and whole-genome sequencing, have facilitated the identification of pathogenic variants in several novel genes associated particularly with autosomal recessive DCM, especially in severe childhood-onset cardiomyopathies. These include genes such as *LEM domain nuclear envelope protein 2* (*LEMD2*), *Acyl-CoA dehydrogenase very long chain* (*ACADVL*), *Cyclase associated actin cytoskeleton regulatory protein 2* (*CAP2*), *TATA-Box binding protein associated factor 1* (*TAF1A*), and *phosphopantothenoylcysteine synthetase* (*PPCS*). These identifications of novel genes and mechanisms have significantly expanded our understanding of the pathophysiological processes underlying DCM and contributed to increasing the yield of genetic testing for both familial and sporadic cases of DCM [47–52]. As an example, we recently identified biallelic pathogenic variants in the *ribosomal protein L3 like* (*RPL3L*) gene as cause of an early-onset, rapidly progressive neonatal DCM and heart failure [53–56]. RPL3L is a paralog of RPL3, a highly conserved and ubiquitously expressed ribosomal protein that forms a component of the 60S ribosomal subunit [57]. Within the ribosomal complex, RPL3 is the ribosomal protein most closely located to the peptidyl transferase center, adjacent to the A-site tRNA binding pocket [58]. Its paralog RPL3L is specifically expressed in skeletal muscle and heart tissue [59, 60]. Interestingly, *RPL3L* mRNA levels exhibit dynamic regulation in response to external stimuli, with a specific downregulation in skeletal muscle cells upon hypertrophic stimuli, and it was proposed that RPL3L might act as a negative regulator of muscular growth [61]. In contrast to its paralog *Rpl3*, homozygous knockout of *Rpl3l* in mice does not result in early embryonic lethality [62, 63]. *Rpl3l*-deficient mice were born at the expected Mendelian ratios and exhibited no significant differences in overall body weight or skeletal muscle/heart weight and did not show signs of cardiac fibrosis [62]. Nevertheless, *Rpl3l*-deficient mice showed a reduced left ventricular ejection fraction, suggesting diminished cardiac contractility [62]. Notably, similar to the observation in C2C12 myogenic cells, the knockout of *Rpl3l* resulted in a compensatory upregulation of *Rpl3* expression in both cardiac and skeletal muscle cells, leading to an elevation of Rpl3 protein levels within ribosomes [61]. The mutational spectrum of *RPL3L* in humans predominantly comprises missense variants; however, compound heterozygosity for truncating or frameshifting variants has also been reported [55, 56]. All patients present with severe, neonatal DCM and rapidly progressing cardiac decompensation leading to early cardiac failure, and patients depend on cardiac transplantation for survival. Pathogenic missense variants are distributed throughout the entire protein, affecting highly conserved amino acids that are located in regions directly involved in RNA binding or interactions between RPL3L and other ribosomal proteins, consequently causing structural perturbations within the 60S ribosomal subunit. Interestingly, heterozygous variants in *RPL3L* have also been linked to atrial fibrillation in humans. Using large-scale data from Iceland and the UK Biobank, it was demonstrated that specific low-frequency coding variants in RPL3L elevate the risk of atrial fibrillation, which could also been observed in distinct DCM patients with biallelic pathogenic variants in *RPL3L*, highlighting the crucial role of RPL3L in cardiac-related processes [45, 64]. Utilizing multi-omics approaches on iPSC-derived cardiomyocytes and corresponding engineered heart muscle (EHM) models of distinct sets of *RPL3L* variants, we are currently elucidating in depth the molecular consequences of specific causative variants and unraveling the underlying mechanisms by which these variants lead to dilated cardiomyopathy.

**Figure 2:**
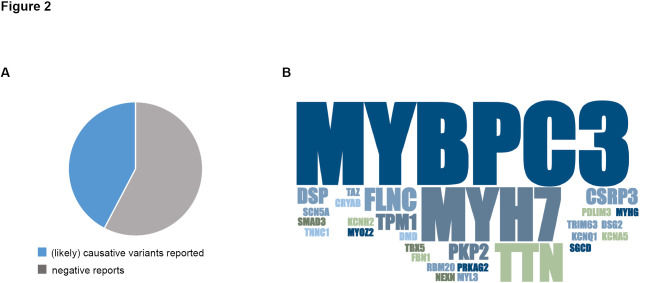
**Diagnostic yield of multigene panel diagnostic for cardiomyopathies at the Institute of Human Genetics in Göttingen over the past five years.** **(A)** Pathogenic or likely pathogenic variants were detected in approximately 40 % of patients who underwent multigene panel testing for inherited cardiomyopathies. **(B)** Overview of genes within the multigene panel in which pathogenic/likely pathogenic variants could be identified. The majority of disease-causing variants were identified in *MYBPC3*, *MYH7*, *TTN*, and *FLNC*.

## Genetic testing of patients with CMs: the missing heritability and future directions

Genetic testing for cardiomyopathies has become an increasingly crucial component of clinical care, and NGS-based advances in sequencing technologies have significantly improved genetic testing as well as data interpretation and classification of detected variants in hereditary CMs. Still, despite improved diagnostic testing and ongoing research efforts, a genetic yield gap persists in both sporadic and specifically familial forms of CM. Moreover, the interpretation of genetic results is further complicated by variable penetrance and expressivity prevalent in CMs. Hereditary cardiomyopathies frequently display significant phenotypic variability, and the penetrance of pathogenic variants is highly variable and typically below 100 %. Overall, modern genetic testing possesses the capability to identify causative variants in up to 50 % of patients with HCM, whereas causative genetic variants can be only detected in approximately 30 % of patients with familial DCM and even less in DCM patients without a recognized family history [65]. The reported detection rates align with the findings observed at the Institute of Human Genetics in Göttingen. Over the past five years, our laboratory has identified and reported pathogenic or likely pathogenic variants in approximately 40 % of patients who underwent multigene panel testing for inherited cardiomyopathies (Figure 2A). The majority of disease-causing variants were identified in *MYBPC3*, *MYH7*, *TTN*, and *FLNC* (Figure 2B). Closing the diagnostic yield gap and identifying causative variants in remaining CM cases is challenging, as these are typically associated with sporadic CM or occur in small families that limit extensive phenotyping and co-segregation analyses. Currently, genetic testing for inherited CMs is performed using targeted multigene panels or whole exome sequencing. However, the interpretation of results can be complicated due to the detection of numerous VUS.

In addition to identifying novel genetic factors associated with CMs, the large-scale generation of whole-genome data from CM patients may be valuable in the future, enabling the determination of e.g. polygenic inheritance and the establishment of so called polygenic risk scores (PRSs). Recent genetic studies have identified a growing number of patients with di- and polygenic inheritance patterns, indicating that multiple high-impact variants can have cumulative effects on disease penetrance and phenotypic outcome [66]. Similar to other diseases such as cancer, PRSs comprising hundreds of single nucleotide polymorphisms (SNPs) are currently being employed to enhance our understanding of the penetrance of monogenic variants leading to CMs, possibly enabling the identification of high-risk individuals in the future [67]. Although PRSs have not yet been approved for clinical application in CMs, they hold the potential to assist in identifying individuals at risk of developing heart failure and, potentially, predicting specific therapeutic responses that could enhance the treatment of genetic cardiomyopathies. Furthermore, the future implementation of long-read sequencing approaches in routine genome diagnostics of CMs will enhance the diagnostic yield, particularly in detecting structural variants in the genome and causative variants in repetitive structures.
